# Optimization of High-Dimensional Functions through Hypercube Evaluation

**DOI:** 10.1155/2015/967320

**Published:** 2015-08-03

**Authors:** Rahib H. Abiyev, Mustafa Tunay

**Affiliations:** ^1^Applied Artificial Intelligence Research Centre, Near East University, P.O. Box 670, Lefkosa, Northern Cyprus, Mersin 10, Turkey; ^2^Computer and Instructional Technologies Education, Eastern Mediterranean University, Famagusta, Northern Cyprus, Mersin 10, Turkey

## Abstract

A novel learning algorithm for solving global numerical optimization problems is proposed. The proposed learning algorithm is intense stochastic search method which is based on evaluation and optimization of a hypercube and is called the hypercube optimization (HO) algorithm. The HO algorithm comprises the initialization and evaluation process, displacement-shrink process, and searching space process. The initialization and evaluation process initializes initial solution and evaluates the solutions in given hypercube. The displacement-shrink process determines displacement and evaluates objective functions using new points, and the search area process determines next hypercube using certain rules and evaluates the new solutions. The algorithms for these processes have been designed and presented in the paper. The designed HO algorithm is tested on specific benchmark functions. The simulations of HO algorithm have been performed for optimization of functions of 1000-, 5000-, or even 10000 dimensions. The comparative simulation results with other approaches demonstrate that the proposed algorithm is a potential candidate for optimization of both low and high dimensional functions.

## 1. Introduction

One of the basic problems of numerical optimization techniques is the computing globally optimal solutions of high-dimensional functions. The aim of optimization is the finding of optimum values of the objective function through learning the parameters of the function given in the defined domains. The learning algorithms are basically divided into two categories. The algorithms based on derivatives of the cost functions (or objective functions) are called derivative based learning algorithms, and the algorithms that do not use the derivatives of the cost functions are called derivative free learning. Recently various learning techniques have been applied to obtain the solution of different optimization problems. However, derivative based learning techniques do not fare well for finding global optimal solutions of the nonlinear problems having many local optimal solutions. Derivative free learning techniques and evolutionary computing are effective optimization techniques that can be used to solve “local minima” problem and find global optimum of the problem.

In the literatures, various learning algorithms have been applied to find global optimal solution. Monte-Carlo method [1], Vegas algorithm [2], and Cat algorithm [3] are extensively used for solution of different optimization problems. Some of more used algorithms are genetic algorithms (GA) [4, 5], evolution strategies [6], differential evolution (DE) [7], particle swarm optimization [8], and other nonevolutionary methods such as simulated annealing [9], tabu search [10], ant-colony optimization (ACO) [11], and artificial bee colony algorithm [12]. The integration of the methods with computational intelligence techniques is widely used to solve different practical problems of engineering and science [13–18].

Recently number of researches has been done on global optimization, but there are still not many powerful techniques for optimization of dense high-dimensional problems. This is because the global optimization of high-dimensional functions is computationally expensive, cost involved. These problems are characterized by many parameters, and many iterations and arithmetic operations are needed for evaluations of these functions. In practical applications, evaluation of the function is often very expensive and large number of function evaluations might not be very feasible [19].

Some learning algorithms have been designed for global optimization of high-dimensional functions. Reference [20] uses new variant of differential evolution (DE), named DECC-I and DECC-II for high-dimensional optimization (up to 1000 dimensions). The algorithms use several novel strategies that focus on problem decomposition and subcomponents cooperation. An improved differential evolution algorithm [21], self-adaptive differential evaluation algorithm [22], differential ant-stigmergy [23], particle swarm optimization [24, 25], modified multiscale particle swarm optimization [26], surrogate-assisted evolutionary programming [27], and group search optimizer (GSO) inspired by animal behavior [28] are designed and applied for global optimization of high-dimensional functions. As shown the designed algorithms are basically modification, improvement, and adaptation of existing evolutionary algorithms in particularly DE, PSO, and GA. Using these methods the researchers try to obtain reasonable results for optimization functions. In spite of some success, these techniques are still not very much suitable for high-dimensional global optimization problems [19]. The proposed algorithms are more suitable for low-dimensional problems. The dimension that was used in above research papers was maximum 100 and some of them 1000. In this paper, the novel method that solves high-dimensional global optimization problems having sizes of 1000, 5000, and 10000 is proposed. The proposed novel method is called hypercube optimization (HO) algorithm. The HO algorithm is based on designing hypercube, selecting the best elements and applied them to multivariate systems for optimization of the objective function. This algorithm approaches optimal points using the best elements determined during learning.

The paper is organized as follows.  Section 2 presents the hypercube optimization algorithm proposed. The processes used in the algorithm are described.  Section 3 describes the test functions used in simulations.  Section 4 includes application of the algorithm on test functions.  Section 5 presents comparative results of HO algarithm with some existing methods. Finally, in Section 6 conclusions are presented.

## 2. Hypercube Optimization Algorithm

The HO algorithm is an evolutionary algorithm that takes inspiration from the behaviour of a dove discovering new areas for food in natural life. In such behaviour a flying dove searches for new locations of food. The dove flies down in a unique way and marks the area that may have food. The dove flies up again and it chooses the previously marked areas and changes and shrinks the sizes of the search area. In a search process, the dove is not limited to a single area. The dove picks new search area according to the density of food (domain for the objective function). The dove stops flying and keeps in mind the area which has food. After eating the food, the dove is looking for a new search area. The dove jumps or flies down another area branch to find a new area. The dove does not fly to another area when it gets to an area that has the most food.

In the paper, the hypercube is used to describe the search area. Inside the search area, the value of an objective function is evaluated according to the quantity and density of food. Next, the functional distances between each of two solutions are determined. This distance helps the algorithm to determine the next new search area. This is performed using the displacement-shrink process.

The hypercube optimization algorithm is a derivative-free learning method based on evaluation of set of points randomly distributed in an *m*-dimensional hypercube. After evaluation the point shifts and contracts according to the average between previous best points in order to determine new best points inside the hypercube. The contraction is greater when the movement is smaller to accelerate the convergence. This operation will be reported as an optimal solution at the end of the iterations.

**Figure 1:**
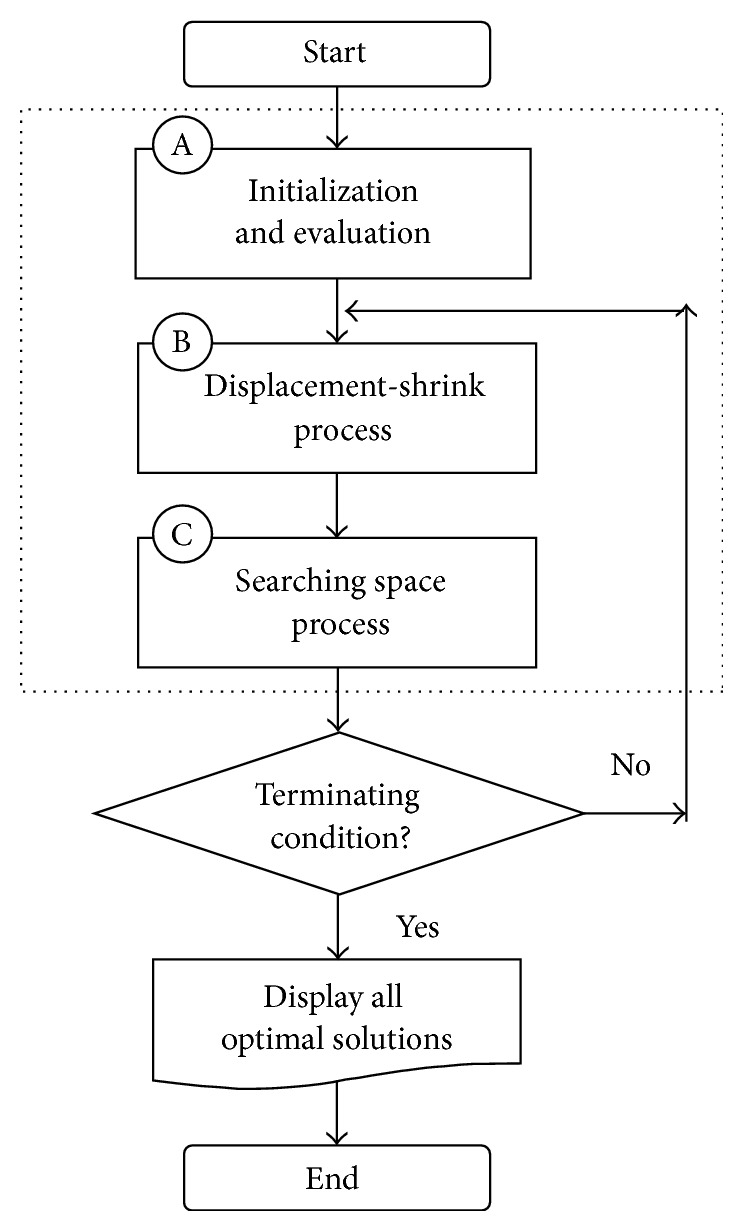
Flowchart of the hypercube optimization algorithm. Here A is* initialization and evaluation*, B is* displacement-shrink*, and C is* searching space* processes.

The HO algorithm is an intense stochastic search method based on hypercube (HC) evaluation. The general structure regarding the visualization of the flowchart of the hypercube optimization algorithm is illustrated in Figure 1. As shown from the figure, the HO algorithm includes three basic processes.

*Step A (initialization and evaluation process).* The algorithm begins with the generation of a hypercube and initialization of matrices and variables within the hypercube. Here the hypercube is represented by the center and size (radii). The new points with uniform distribution are randomly generated within the hypercube. It proceeds to the through main loop, by which convergence to the global minimum is sought, and it finishes when any of the termination criteria is fulfilled.

*Step B (displacement-shrink process)*. The displacement-shrink process is deployed to find the new best point. This is implemented by computing the average of the current best point and the previous best one. The average between both values is taken as a conservative measure to avoid excessive fluctuations in the search.

*Step C (searching space process)*. The searching space process controls the movements of *X* solutions according to the defined interval (commonly [0,0.1]). The searching space process initializes a new hypercube and repeats the whole process.

The initialization and evaluation process, displacement-shrink process, and searching space process are repeated in each learning iteration. While specific termination conditions are satisfied the whole processes are continued to execute.

At each iteration, the newly generated hypercube changes and shrinks its sizes until the optimum points are located. Unlike other methods, like particle swarm optimization, the points in the hypercube optimization algorithm do not move according to a specific rule nor does the method record them, except for the best points. This permits a rapid selection of a new best zone and an intense search in it. Thus, the hypercube optimization algorithm does not perform any local search but rather it is always global. This behavior allows the algorithm to move rapidly to globally best points, as it does not waste time in local searches.

Following in the next subsections the descriptions of each step are presented in detail.

### 2.1. Hypotheses and Representation of Solution

As in all real-valued single-objective unconstrained optimization algorithms, we try to find the minimum (or equivalently the maximum) of a scalar objective function *f*_(*x*)_ and represent the free parameters as a vector or point *X* = (*x*_1_, *x*_2_, *x*_3_,…, *x*_*m*_), where *m* is the dimension of the problem. Therefore, *f* is a mapping *ℜ* ^*m*^ → *ℜ*. We assume the following hypotheses.

(i) *f* is available only as a black box; that is, we have no knowledge or possibility of control of its interior functions. We access *f* only via input-output.
(ii) *f* has a continuous domain inside the bounds; that is, every point inside the bounds has a mapping by *f*.
(iii) *f* is well-behaved in the domain, at least numerically; that is, it is continuous and presents certain smoothness. This constrains overly noisy functions, where there is no spatial correlation. But implicit is also the assumption of some noisiness, whereby finite differences in the neighborhood of a point are not similar to the derivatives of the noiseless function.
(iv) The number of searching points (*N*) is enough for correctly sampling *f*'s domain (related to the previous point). Therefore, *N* is directly related to the dimension of the problem (*m*) and *f*'s smoothness.

### 2.2. Initialization and Evaluation Process

Initialization and evaluation is the first block of hypercube optimization algorithm. The starting conditions are

(1) initial (and global) boundaries for all points: these boundaries are the sides of the hypercube;
(2) initialization of solutions inside the hypercube and an initial random choice of a best point *X*_0_ (if not available, the central point of the initial hypercube will be taken) in the given set.

Initial points of the hypercube optimization algorithm are presented in Table 1. At the starting stage the data radii and centre of the HC are generated randomly and these parameters are used to initialize the first HC. Then uniformly distributed *N* searching points are generated inside the hypercube. Using these points, the values of the objective function are determined. Here the concept is to have an approximate knowledge about the location of the lowest values of *f*. This initial sampling has to be sufficiently dense so as to probe all the possible zones of higher and lower values; otherwise, the algorithm can take the zone sought (global optimum) as a simply better one (local optimum). As pointed out above, this density (and hence the number of points *N*) is a function of the dimension *m* and the smoothness of the function. The problems with higher dimension will require higher *N*.

**Figure 2:**
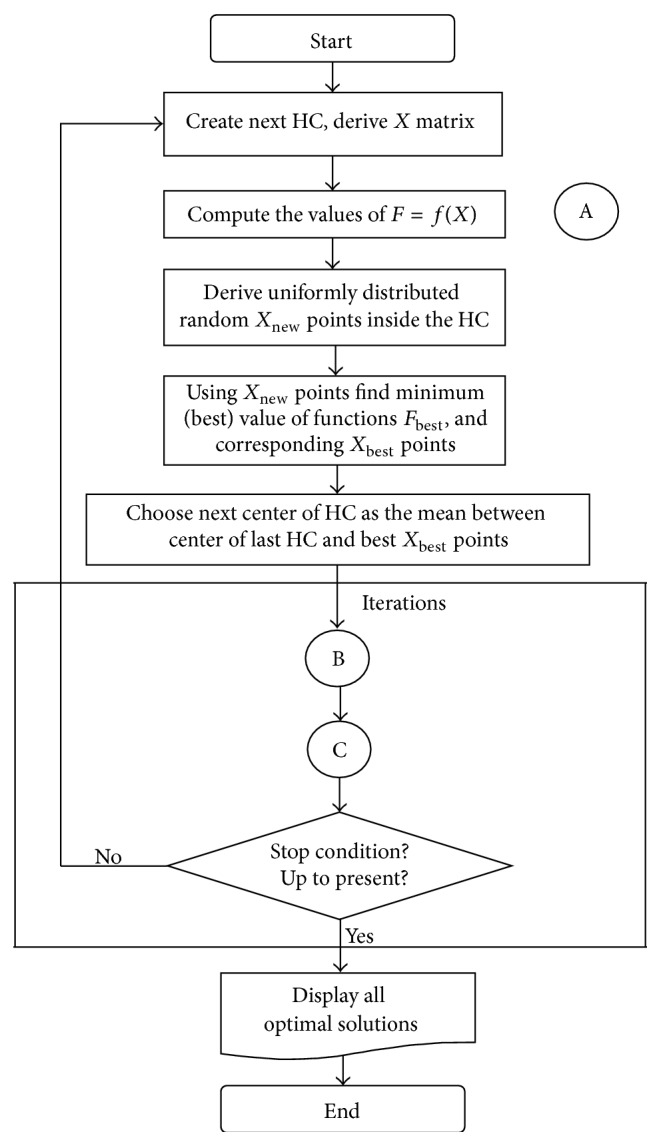
Flowchart of the initialization and evaluation process. Here B is displacement-shrink process and C is searching space process.

The hypercube optimization algorithm begins with the initialization of matrices and variables; it proceeds to the main loop, by which convergence to the global minimum is sought, and it finishes when any of the termination criteria is fulfilled. The details regarding the visualization of the flowchart initialization and evaluation of the HO algorithm are illustrated in Figure 2. After the start block, initial point *X*_0_ is generated as the centre of the first hypercube (HC). The initial value of the radii of the first HC is determined according to the change interval of the test (objective) functions. Next using the value of centre *X*_0_ the dimension of the hypercube is derived according to formula (1). After creating the hypercube, the *X* matrix is generated within this hypercube. The size of *X* is defined by (*N* × *m*). *N* is a number of generated points. We need to comment that in future iterations (*i* = 2,3,…) the hypercube is created using the values of *X* matrix.

We have illustrated this process as follows with initial points to create them with default values.

(1) Dimension of hypercube is
  (1) $$\begin{array}{c} m=\text{length}\left(X_{0}\right). \end{array}$$
(2) Row vectors with lower and upper boundaries of HC are
  (2) $$\begin{array}{c} \text{LB}=\min\left(X\;\text{bounds}\right),\\ \text{UB}=\max\left(X\;\text{bounds}\right). \end{array}$$
(3) Dimensions of *m*-dimensional HC's are
  (3) $$\begin{array}{c} D=\text{UB}-\text{LB}. \end{array}$$
(4) Central values are
  (4) $$\begin{array}{c} X_{c}=\frac{\left(\text{LB}+\text{UB}\right)}{2}. \end{array}$$
(5) Vector with radii of HC is
  (5) $$\begin{array}{c} R_{0}=\frac{D}{2},\\ R=R_{0}. \end{array}$$

According to *X* matrix, the row vector with lower and upper boundaries of the hypercube (2) is determined. Using these boundaries, obtained from the first hypercube (zone), the radii (4) and the centre (5) points of the next hypercube are determined. *X* matrix, defined as *N* searching points, is applied to determine the values of the test function, that is, *F*(*f*(*x*)) matrix, as pointed out above in Table 1. In the next step using the HC, the new uniformly random points are derived. The number of points is defined according to the dimension of the HC. These points form the new *X*_new_ matrix. This matrix is used to evaluate the test functions. As a result of evaluation, the best (minimum) value of function *F*_best_ and the corresponding *X*_best_ points are determined. By “best” we mean the vector that corresponds to the best fitness (e.g., the lowest objective function value for a minimization problem) in the entire population at *i*th iteration. The *X*_best_ point is improved (updated) using local search; that is, *X*_best_^new^ = *X*_best_ + *ρ*Δ*F*. Here 0 ≤ *ρ* ≤ 1, *F* is the objective function. The improvement is continued until Δ*F* becomes acceptably small value less than a preset value (tol*F*). The derived best points are used to determine the centre and the radii of the next hypercube. This operation is realized by calculating the mean of the center of the last HC (*X*_last_centre_) and the previous best (*X*_best_) points; that is, (*X*_last_centre_ + *X*_best_)/2. This process is called “displacement.” As shown the created second HC is derived from the previous HC and the sizes of the second HC will be less than the sizes of the previous one. In future operations, the last-second HC will be used to create the next-third hypercube.

In summary, we can unify the evaluation and learning processes as follows. When the new hypercube is initialized, the function is evaluated at new points, randomly (with uniform distribution) chosen from inside of the hypercube. The new minimum is determined and compared with the last minimum. If the new minimum is worse (greater) than the previous one, then a new iteration will be started. If the same value is repeated several consecutive times then the algorithm ends, and the best minimum is considered as the global minimum.

After the above given initialization and evaluation processes the implementation of displacement-shrink process and searching space process is performed. The whole process is repeated until specific termination conditions are satisfied.

### 2.3. Displacement and Shrink Process

The center of the next hypercube will be just the average between the current best point and the previous one; that is, (*X*_last_centre_ + *X*_best_)/2. The average between both values is taken as a conservative measure to avoid excessive fluctuations in the search and to prevent moving suddenly to a neighboring zone where a lower value was found, but which perhaps is just a local minimum. The radii of the new hypercube are determined as *R*_new_ = *R*_old_∗*S*. Here *S* is a factor of convergence which is defined in the next section (see (10)).

In addition to moving, the hypercube has to contract in order to refine the search and to converge to a unique and certain—assumed global—minimum. This contraction is controlled by the movement of the average of best values. For large displacements, there is no contraction, as we interpret that the global minimum is still very uncertain. For small or null displacements, the hypercube will shrink, as we interpret this to mean that we are closer to the global minimum: the contraction is greater for smaller movements. This derives the fast convergence of the method, while it prohibits getting stuck at undesired (local) minima.

**Figure 3:**
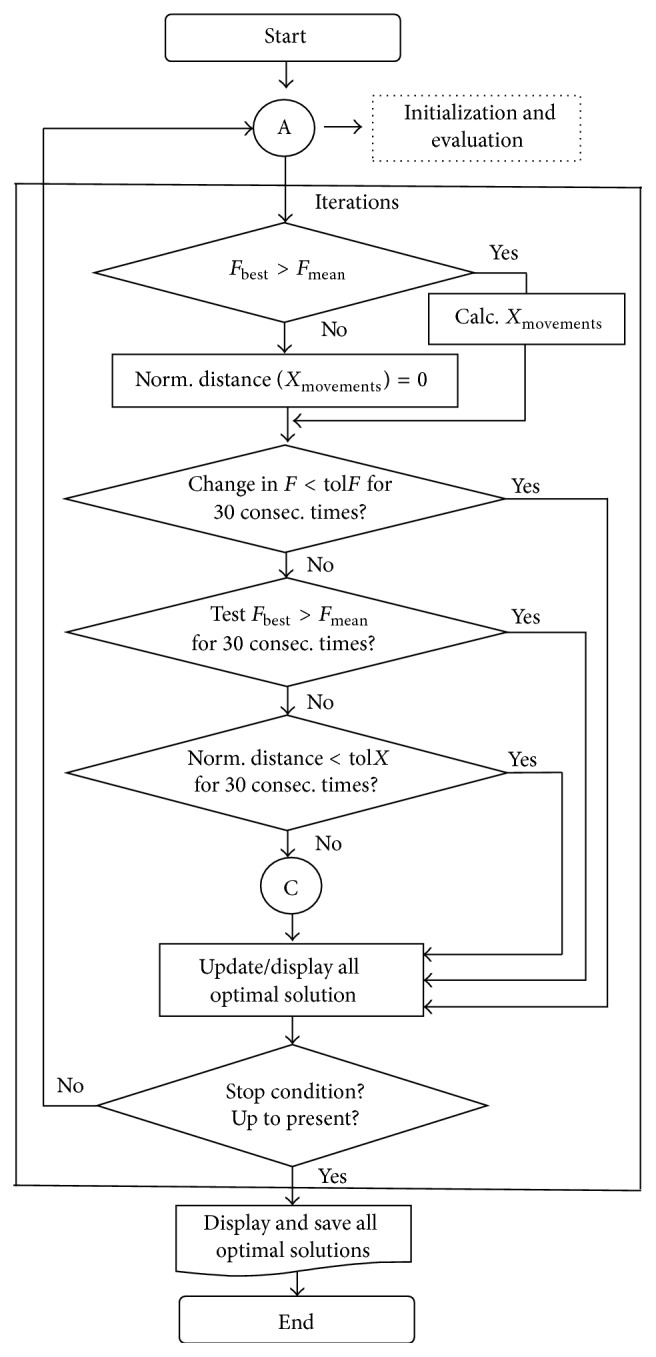
Flowchart of the displacement-shrink process. Here A is initialization and evaluation process and C is searching space process.

The details regarding the visualization of the flowchart of the displacement-shrink process of the hypercube optimization algorithm is illustrated in Figure 3. At first, the minimum of value of *F*_best_ is compared with the new value of *F*_mean_ corresponding to the point mean = (*X*_last_centre_ + *X*_best_)/2 determined as pointed out in the previous section. If *F*_mean_ value is less than *F*_best_ value then, in given iteration, *X* displacement (or *X* movement) is computed and normalized twice: first each element of *X* is divided by the corresponding initial range (and thus the displacement is transformed into a unity-sided hypercube) and then that quantity is normalized again, dividing it by the diagonal of hypercube $\sqrt{m}$. These operations are illustrated as follows:

(1) normalized *X*_*n*_ (previous *X* for minimum):
  (6) $$\begin{array}{c} X_{n}=\frac{\left(X-X_{c}\right)}{D}, \end{array}$$
(2) normalized *X*_min⁡_ (current *X* for minimum):
  (7) $$\begin{array}{c} X_{\min n}=\frac{\left(X_{\min}-X_{c}\right)}{D}, \end{array}$$
(3) normalized distance (should be bounded by 0 and sqrt of *m*):
  (8) $$\begin{array}{c} d_{n}=\frac{\text{sum}{\left({\left(X_{n}-X_{\min n}\right)}^{2}\right)}^{0.5}}{D}, \end{array}$$
(4) renormalized distance (should be bounded by 0–0.1):
  (9) $$\begin{array}{c} d_{nn}=\frac{d_{n}}{\sqrt{m}}. \end{array}$$

In the result of these operations, *X*_*n*_ points are shrunk (become smaller) to the centre point *X*_*c*_. These points are used to evaluate the test functions again. In the next blocks the hypercube continues moving and shrinking until one of the following conditions are not met.

(i) The change in consecutive *F*_best_ values is smaller than a preset value (tol*F*), for a preset consecutive number of times. This is also interpreted as convergence in *F* space.
(ii) The same or worse *F* value is found consecutively a preset number of times. This is interpreted as nonconvergence in *F* space.
(iii) The change in best *X* value (renormalized distance) is smaller than a preset value (tol*X*), for a preset consecutive number of times. This is interpreted as convergence in *ℜ* ^*m*^ space. The whole process is repeated until specific termination conditions are satisfied.
(iv) The maximum number of iterations is reached: of course, in this case convergence is not guaranteed, as possibly lower values could be found with more iteration.

Each condition is tested for thirty consecutive times. If these conditions are not satisfied then the searching space process will be initialized.

We need to notice that the movement of *X* will not be performed if the *F*_mean_ value will be larger than *F*_best_ value. In such case, the searching space process will be initialized.

### 2.4. Searching Space Process

**Figure 4:**
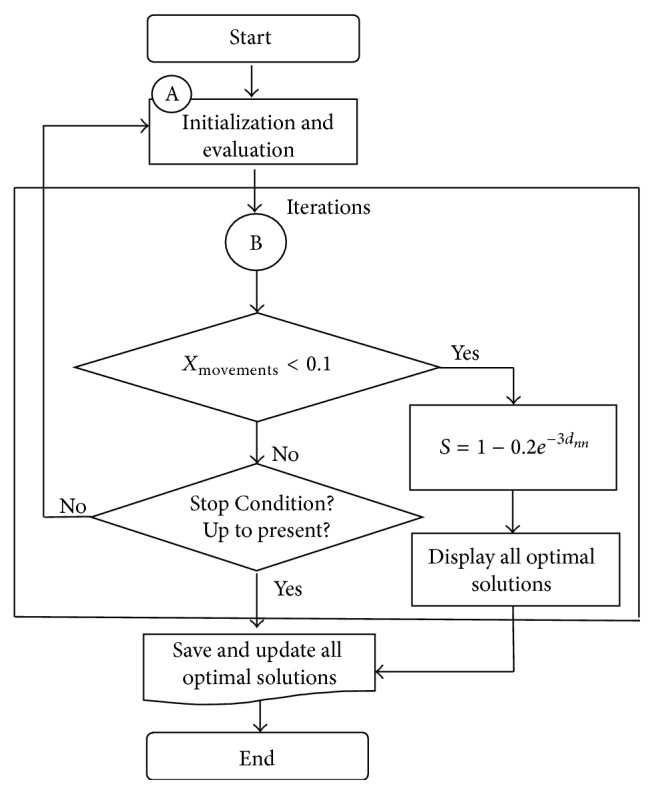
Flowchart of the searching space process. Here A is initialization and evaluation process and B is displacement-shrink process.

The searching space process initializes new center and size (radii) in order to create new hypercube. The objective function is evaluated at new points which are randomly chosen from the hypercube and having uniform distribution. The searching space process controls the movements of *X* according to the interval defined, in particularly for *X*_movements_ < 0.1. The value of *X*_movement_ is determined by *d*_*nn*_. The flowchart of the searching space process of the HO algorithm is illustrated in Figure 4.

If the movement of *X* satisfies the condition then a factor of convergence *S* is calculated and updated at each iteration:

(10) $$\begin{array}{c} S=1-0.2e^{-3d_{nn}}, \end{array}$$

where *d*_*nn*_ is computed by (9) and describes the normalized distance moved by the average of last two best values of *X*. Next the update of solutions will be performed. The size (in all the dimensions) of the hypercube is reduced by multiplying by this factor. Thus, the hypercube reduces or maintains its size for nontrivial movements and shrinks otherwise. The whole process is repeated until specific termination conditions are satisfied.

## 3. Test Functions

The proposed hypercube optimization algorithm is tested on five continues test functions which are widely used in the literatures:* Ackley path function*,* Rastrigin function*,* Rosenbrock function*,* Griewank function,* and* Sphere function* [19–23]. The test functions are more applicable for the experimental evaluations of methods used in global optimization problems. The designed algorithm is implemented in MATLAB.

### 3.1. Ackley Path Function

*Ackley path function* is continuous, scalable, and nonseparable and is an extensively multimodal test function.

This test function is formulated as follows:

(11) f1x=−20exp⁡⁡−0.21D∑i=1Dxi2 −exp⁡1D∑i=1Dcos⁡2πxi+20+e,

where *D* is a number of dimensions and *x*_*i*_ = (*x*_1_, *x*_2_,…, *x*_*D*_) is *D* dimensional row vector. The test area is usually evaluated in the interval of −32 ≤ *x*_*i*_ ≤ 32, *i* = (1,…, *D*). Global minimum *f*(*x*) = 0 is obtainable for *x*_*i*_ = (0,0).

### 3.2. Rastrigin Function

*Rastrigin function* is continuous, scalable, and separable and is highly multimodal global optimization function.

This test function is formulated as follows:

(12) $$\begin{array}{c} f_{2}\left(x\right)=10D+\overset{D}{\underset{i=1}{\sum }}\left({x_{i}}^{2}-10\cos\left(2\pi x_{i}\right)\right), \end{array}$$

where *D* is a number of dimensions and *x*_*i*_ = (*x*_1_, *x*_2_,…, *x*_*D*_) is *D* dimensional row vector. The test area is usually evaluated in the interval of −5.12 ≤ *x*_*i*_ ≤ 5.12, *i* = (1,…, *D*). Global minimum *f*(*x*) = 0 is obtainable for *x*_*i*_ = (0,0).

### 3.3. Rosenbrock Valley Function

*Rosenbrock's valley function* is known as the* second function of De Jong*. This test function is continuous, scalable, naturally nonseparable, nonconvex, and unimodal.

This test function is formulated as follows:

(13) $$\begin{array}{c} f_{3}\left(x\right)=\overset{D-1}{\underset{i=1}{\sum }}\left[100{\left(x_{i+1}-{\left(x_{i}\right)}^{2}\right)}^{2}+{\left(x_{i}-1\right)}^{2}\right], \end{array}$$

where **D** ≥ 2 is a number of dimensions and *x*_*i*_ = (*x*_1_, *x*_2_,…, *x*_*D*_) is *D* dimensional row vector. The test area is usually evaluated in the interval of −2.048 ≤ *x*_*i*_ ≤ 2.048, *i* = (1,…, *D*). Global minimum *f*(*x*) = 0 is obtainable for *x*_*i*_ = (1,1).

### 3.4. Sphere Function

The simplest benchmark function is* sphere model* which is also called De Jong's function 1. This test model is continuous, unimodal, and appearance of convex.

This test function is formulated as follows:

(14) $$\begin{array}{c} f_{4}\left(x\right)=\overset{D}{\underset{i=1}{\sum }}{x_{i}}^{2}, \end{array}$$

where *D* is a number of dimensions and *x*_*i*_ = (*x*_1_, *x*_2_,…, *x*_*D*_) is a dimensional row vector. The test area is usually evaluated in the interval of −5.12 ≤ *x*_*i*_ ≤ 5.12, *i* = (1,…, *D*). Global minimum *f*(*x*) = 0 is obtainable for *x*_*i*_ = (0,0).

### 3.5. Griewank Function

*Griewank function* is continuous, scalable, nonseparable, and multimodal test function.

This test function is formulated as follows:

(15) $$\begin{array}{c} f_{5}\left(x\right)=\frac{1}{4000}\overset{D}{\underset{i=1}{\sum }}{x_{i}}^{2}-\overset{D}{\underset{i=1}{\prod }}\cos\left(\frac{x_{i}}{\sqrt{i}}\right)+1, \end{array}$$

where *D* is a number of dimensions and *x*_*i*_ = (*x*_1_, *x*_2_,…, *x*_*D*_) is a dimensional row vector. The test area is usually evaluated in the interval of −600 ≤ *x*_*i*_ ≤ 600, *i* = (1,…, *D*). Global minimum *f*(*x*) = 0 is obtainable for *x*_*i*_ = (0,0).

## 4. Simulation Studies

The performance of the hypercube optimization algorithm is tested on the five benchmark functions given above. The benchmark functions *f*_1_ ÷ *f*_5_ are evaluated by considering the cases in which the problem dimensions are set as 1000, 5000, or even 10000 dimensions. At first the dimension is set as 1000. The population size is also set to 100, 1000, or even 10000. We have summarized the best average fitness (e.g., the lowest objective function value) and the average number of the test function evaluations over successful 30 runs. For each evaluation, the learning of the algorithm is continued 5000 iterations. The hypercube optimization algorithm has global minimum that was obtained with much well convergence process for these test functions.

No optimization algorithm guarantees convergence for any function, but it is a good practice to test the HO algorithm for several benchmark functions and tune the parameters.

Therefore, we have tested the hypercube optimization algorithm on a set of benchmark functions, and the algorithm has yielded improved results, sometimes reaching the better solution faster than well-established algorithms. The details regarding the visualization of the test function results are given below.

In the next step, the test functions are evaluated for the cases in which the problem dimensions of *f*_1_ ÷ *f*_5_ are set to 5000 or even 10000 dimensions. The population size is set to 100. The convergence graphics have also been obtained and averaged through evaluations over successful 30 runs. The details of results regarding the visualization of the test function are given as follows.

### 4.1. Ackley Path Function

**Figure 5:**
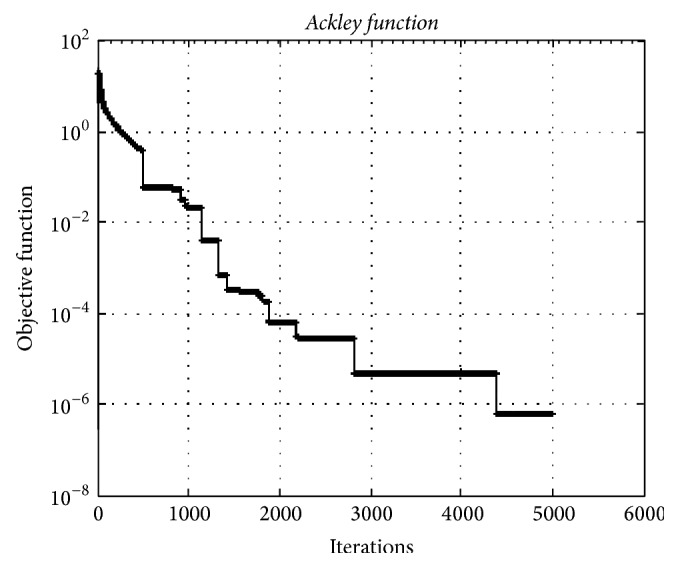
The convergence graphic for* the Ackley function* with dimension of 5000 and population of 100.

*The Ackley path function* is an extensively used multimodal test function.  Figure 5 ilustrates the convergence graphic of HO algorithm for 5000 dimensions. The population size of the HO algorithm is almost insensitive to the dimension of the problems. The minimum of* Ackley* test function was obtained as 2.76*e* − 07.

**Figure 6:**
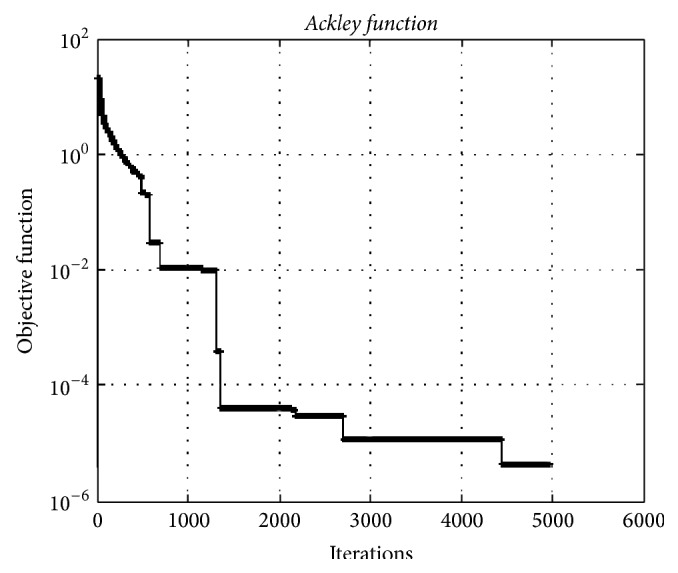
The convergence graphic for the* Ackley function* with dimension of 10000 and population of 100.

Figure 6 depicts the convergence graphic of the HO algorithm for the* Ackley* test function having 10000 dimensions. The minimum value of the function was obtained as 1.16*e* − 06.

### 4.2. Rastrigin Function

The* Rastrigin function* is a typical nonlinear multimodal function. This test function is a fairly difficult problem for evolutinary algorithms due to the high number of dimensions and large number of local minima.

**Figure 7:**
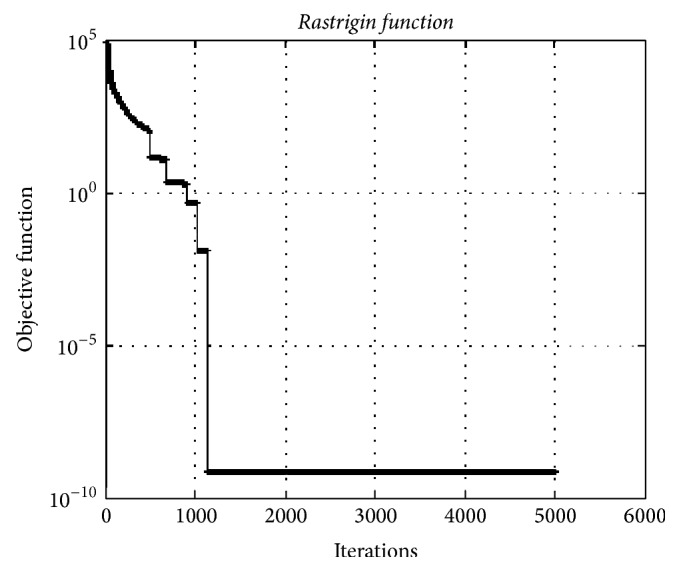
The convergence graphic for the* Rastrigin function* with dimension of 5000 and population of 100.

Figure 7 depicts the convergence graphic of HO algorithm for the* Rastrigin* test function having 5000 dimensions. The minimum was obtained as 7.13*e* − 10. The HO algorithm can find near-optimal solutions with much well convergence with high dimension for this test function.

**Figure 8:**
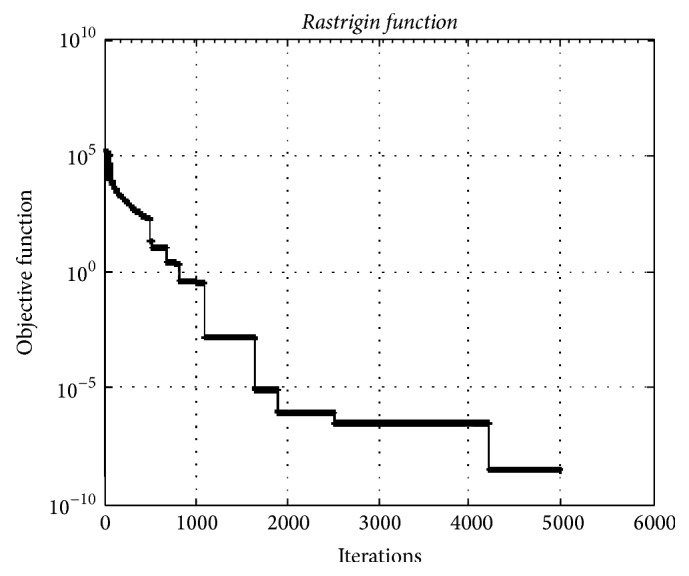
The convergence graphic for the* Rastrigin function* with dimension of 10000 and population of 100.

Figure 8. It depicts the convergence graphic for the test function having 10000 dimensions. The minimum value of function was obtained as 2.99*e* − 09.

### 4.3. Rosenbrock Function

The* Rosenbrock function* is a typical naturally nonseparable, nonconvex, and unimodal. This test function is also a fairly hard problem for evolutionary algorithms.

**Figure 9:**
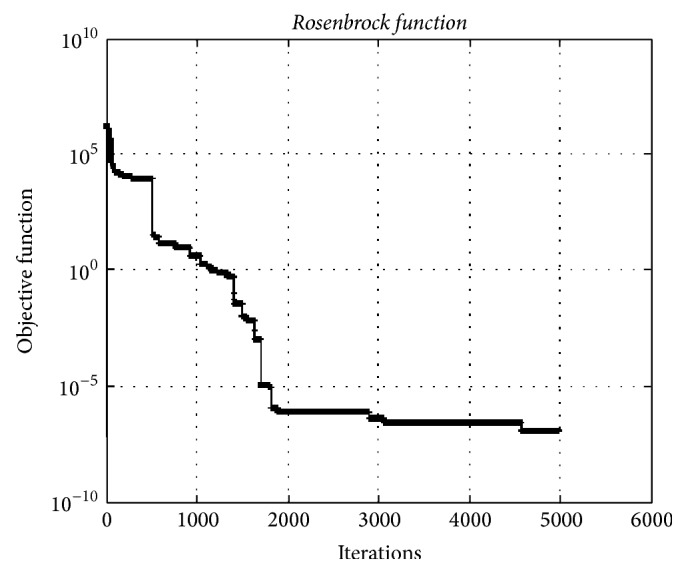
The convergence graphic for the* Rosenbrock function* having dimension of 5000 and population of 100.

Figure 9 depicts the convergence graphic for the* Rosenbrock test function* having 5000 dimensions. The minimum value of function was obtained as 1.15*e* − 08. The HO algorithm can find optimal or near-optimal solutions with much well convergence. This fact indicates that HO algorithm is almost insensitive to the dimension of the problems.

**Figure 10:**
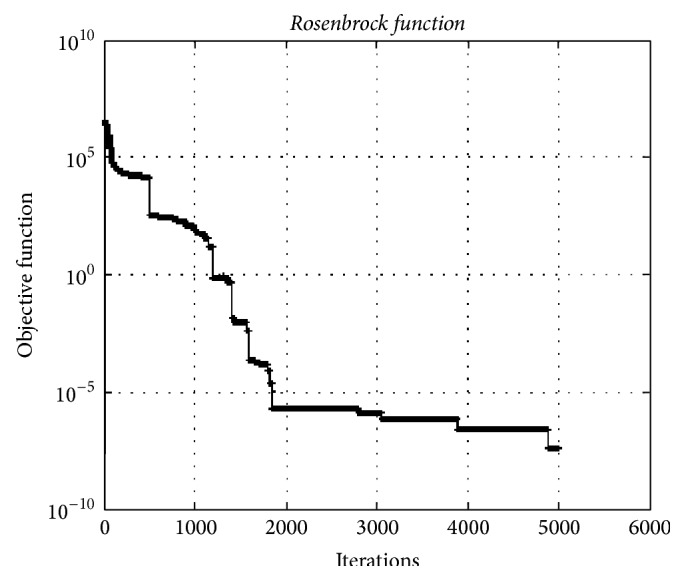
The convergence graphic for the* Rosenbrock function* having dimension of 10000 and population of 100.

Figure 10 depicts the convergence graphic for the* Rosenbrock function* having 10000 dimensions. The minimum value of function was obtained as 3.38*e* − 08.

### 4.4. Sphere Function

**Figure 11:**
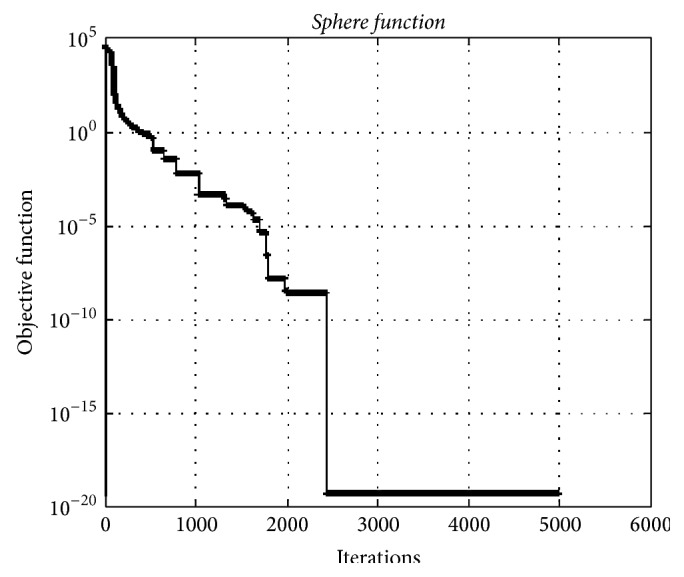
The convergence graphic for the* Sphere function* having 5000 dimensions and 100 populations.

The* Sphere function* is a typical unimodal test function.  Figure 11 depicts convergence graphic of HO algorithm for the* Sphere* test function having 5000 dimensions. The minimum value of test function using HO algorithm was obtained as 4.64*e* − 020.

**Figure 12:**
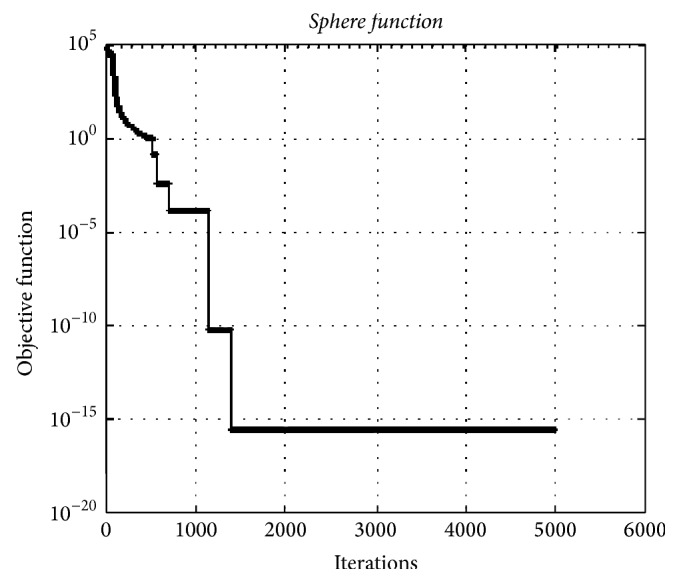
The convergence graphic for the* Sphere function* having 10000 dimensions and 100 populations.

In Figure 12, the convergence graphic of hypercube optimization algorithm for the* Sphere* test function having 10000 dimensions is given. The minimum value was obtained as 2.40*e* − 016 with much well convergence. This test function is a fairly easy problem for finding the total optimum and in the fast convergence.

### 4.5. Griewank Function

The* Griewank function* is also a typical nonlinear multimodal function. This test function is tested using many multiobjective evolutionary algorithms [23].

**Figure 13:**
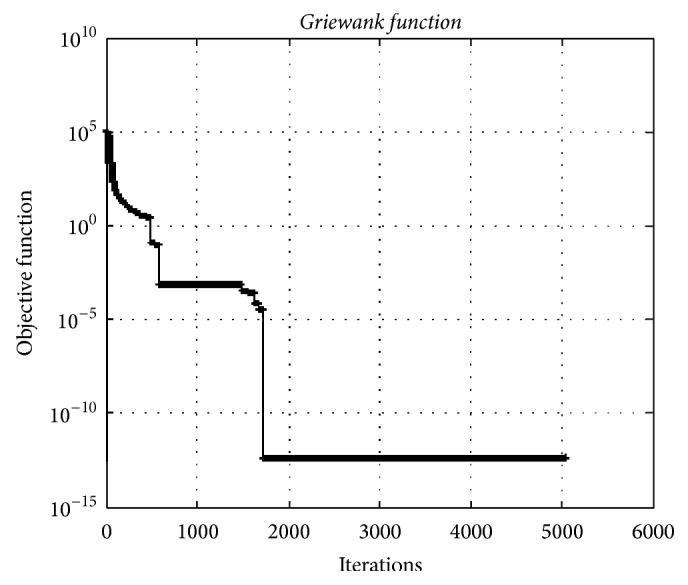
The convergence graphic for the* Griewank function* having dimension of 5000 and population of 100.

Figure 13 depicts the convergence graphic for the* Griewank* test function having 5000 dimensions. The minimum value of function was obtained as 3.34*e* − 013.

**Figure 14:**
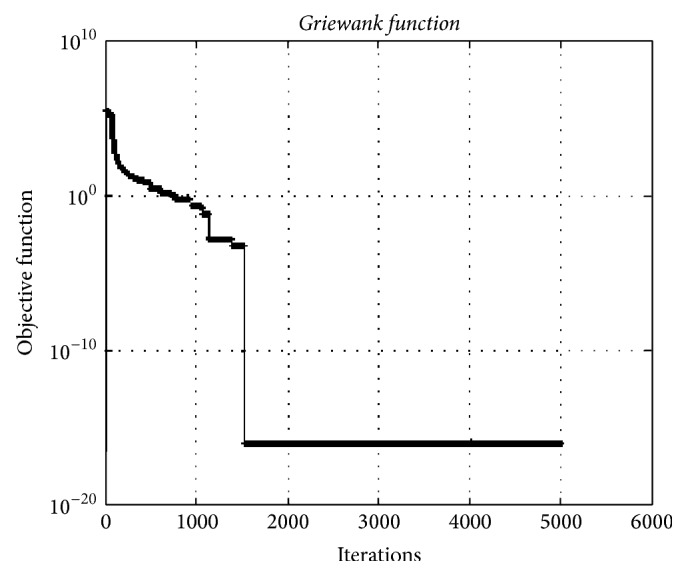
The convergence graphic for the* Griewank function* having dimension of 10000 and population of 100.

In Figure 14, the minimum value of test function using HO algorithm was obtained as 1.11*e* − 016 for 10000 dimensions. The HO algorithm can find optimal or near-optimal solutions with much well convergence with high dimension for this test function.

## 5. Comparison

The hypercube optimization algorithm has yielded in general quite better results, sometimes reaching the better solution faster than well-established algorithms. The usage of multiobjective evolutionary algorithms allows us to find global optimal solutions and avoid local optimum problem.

The simulation results of HO algorithm that was obtained with test functions with different dimensions and averaged over 30 runs are given in Table 2. Using the table we can see that by increasing learning iterations from 1000 to 5000, the performance of HOA is increased for functions *f*_1_, *f*_2_, *f*_3_, and *f*_4_ as 2.46*e* − 013, 4.54*e* − 011, 8.16*e* − 017, 6.32*e* − 015, and 5.86*e* − 059 correspondingly.

This chapter presents comparison of the performances of the hypercube optimization algorithm, with the two popular global optimization approaches, namely, genetic algorithm (GA) and particle swarm optimization (PSO) acting on above given four benchmark functions, namely,* Ackley path function*,* Rastrigin function*,* Rosenbrock function,* and* Sphere function*. These test functions are evaluated by considering the cases in which the problem dimensions of *f*_1_, *f*_2_, *f*_3_, and *f*_4_ are set as *D* = 1000 for the 1000 iterations. The proposed algorithm is tested by using above given test functions and the main unknown parameters are determined. The values of main parameters for GA and PSO used in this chapter can be found in detail in [19, 29].

In Table 3, comparison of all the three algorithms for test functions of 1000 dimensions is provided.

All the algorithms were tested for 1000 dimensions. As evident from the results presented in Table 3, the HO algorithm obtains better results (reflected in the average fitness) than other techniques. The comparative results of the algorithms demonstrate that the performance of HO algorithm improves upon other well-known global optimization techniques: GA and PSO.

## 6. Conclusion

This paper proposes the hypercube optimization algorithm to solve multivariate systems for global optimization. The designed algorithm is based on a hypercube evaluation driven by convergence. The use of stochastic search method approach allows it to speed up the learning of the system and, respectively, to decrease training time of the system with a faster convergence. The simulations have been carried out using benchmarking functions, such as* Ackley function*,* Rastrigin function*,* Rosenbrock function*,* Sphere function,* and* Griewank function*. The computational results have demonstrated that the performance of the system have considerably increased in optimization problems for solving a set of global optimization problems with large numbers of populations. The population size of the HO algorithm is almost sensitive to the dimension of the problems for these test functions. The comparative results of HOA, GA, and PSO algorithms demonstrate that the performance of HO algorithm is an improvement upon other two global optimization techniques.

## Figures and Tables

**Table 1:** Initial points.

Symbol	Definition
*m*	Dimension of hypercube
*R*	radii of HC
*X*_*c*_	Center of 1st HC (zone)
*X* = *X*_0_	Take initial point as 1st HC
LB, UB	Lower and upper bounds of first HC (zone)
*N*	Number of points in each HC
*X*	*N* × *m* points, solutions
*F*	*N* × 1 points, values of functions
*F*_best_	Best value of objective function

Create matrices: *X*(*N* × *m*) *F*(*N* × 1) *F*_best_: best value of objective function	

**Table 2:** Results of the mean best functions values averaged over 30 runs obtained by HO algorithm.

Function	Population	Dimension	The best	Iterations
*f*_1_	100	1000*d*	5.01*e* − 012	5000
	1000	1000*d*	2.46*e* − 013	5000
	10000	1000*d*	5.12*e* − 012	5000

*f*_2_	100	1000*d*	1.83*e* − 010	5000
	1000	1000*d*	4.54*e* − 011	5000
	10000	1000*d*	3.63*e* − 011	5000

*f*_3_	100	1000*d*	5.68*e* − 017	5000
	1000	1000*d*	8.16*e* − 017	5000
	10000	1000*d*	2.06*e* − 017	5000

*f*_4_	100	1000*d*	1.56*e* − 059	5000
	1000	1000*d*	5.86*e* − 059	5000
	10000	1000*d*	1.12*e* − 072	5000

*f*_5_	100	1000*d*	2.22*e* − 015	5000
	1000	1000*d*	6.32*e* − 015	5000
	10000	1000*d*	5.44*e* − 015	5000

**Table 3:** Comparison of the results.

Function	HO algorithm	GA	PSO	Iterations
*f*_1_	1.07*e* − 003	7.87	9.02	1000
*f*_2_	6.07*e* − 004	1.07*e* + 04	1.40*e* + 04	1000
*f*_3_	5.13*e* − 002	1.12*e* + 03	6.58*e* + 06	1000
*f*_4_	1.16*e* − 008	3.45*e* + 03	5.50*e* + 03	1000
